# Use of a Portable Functional Near-Infrared Spectroscopy (fNIRS) System to Examine Team Experience During Crisis Event Management in Clinical Simulations

**DOI:** 10.3389/fnhum.2019.00085

**Published:** 2019-03-05

**Authors:** Jie Xu, Jason M. Slagle, Arna Banerjee, Bethany Bracken, Matthew B. Weinger

**Affiliations:** ^1^Faculty of Science, Center for Psychological Sciences, Zhejiang University, Hangzhou, China; ^2^Center for Research and Innovation in Systems Safety, Vanderbilt University Medical Center, Nashville, TN, United States; ^3^Department of Anesthesiology, Vanderbilt University Medical Center, Nashville, TN, United States; ^4^Charles River Analytics, Cambridge, MA, United States; ^5^Geriatric Research Education and Clinical Center, VA Tennessee Valley Healthcare System, Nashville, TN, United States

**Keywords:** functional near-infrared spectroscopy (fNIRS), neural synchrony, team engagement, workload, clinical simulation

## Abstract

**Objective:** The aim of this study was to investigate the utilization of a portable functional near-infrared spectroscopy (fNIRS) system, the fNIRS Pioneer^TM^, to examine team experience in high-fidelity simulation-based crisis event management (CEM) training for anesthesiologists in operating rooms.

**Background:** Effective evaluation of team performance and experience in CEM simulations is essential for healthcare training and research. Neurophysiological measures with wearable devices can provide useful indicators of team experience to compliment traditional self-report, observer ratings, and behavioral performance measures. fNIRS measured brain blood oxygenation levels and neural synchrony can be used as indicators of workload and team engagement, which is vital for optimal team performance.

**Methods:** Thirty-three anesthesiologists, who were attending CEM training in two-person teams, participated in this study. The participants varied in their expertise level and the simulation scenarios varied in difficulty level. The oxygenated and de-oxygenated hemoglobin (HbO and HbR) levels in the participants’ prefrontal cortex were derived from data recorded by a portable one-channel fNIRS system worn by all participants throughout CEM training. Team neural synchrony was measured by HbO/HbR wavelet transformation coherence (WTC). Observer-rated workload and self-reported workload and mood were also collected.

**Results:** At the individual level, the pattern of HbR level corresponded to changes of workload for the individuals in different roles during different phases of a scenario; but this was not the case for HbO level. Thus, HbR level may be a better indicator for individual workload in the studied setting. However, HbR level was insensitive to differences in scenario difficulty and did not correlate with observer-rated or self-reported workload. At the team level, high levels of HbO and HbR WTC were observed during active teamwork. Furthermore, HbO WTC was sensitive to levels of scenario difficulty.

**Conclusion:** This study showed that it was feasible to use a portable fNIRS system to study workload and team engagement in high-fidelity clinical simulations. However, more work is needed to establish the sensitivity, reliability, and validity of fNIRS measures as indicators of team experience.

## Introduction

### Simulation Training in Healthcare

Simulation is “a technique that uses a situation or environment created to allow persons to experience a representation of a real event for the purpose of practice, learning, evaluation, testing, or to gain understanding of systems or human actions” (Huang et al., 2008). Simulation has been widely used in complex work system domains, such as aviation, ground transportation, process control, military command and control, and healthcare (Rosen, 2008; Vincenzi et al., 2009; Salas and Maurino, 2010; Fisher et al., 2011; Skjerve and Bye, 2011). In healthcare, simulation exists in many different forms, from verbal simulations with role-playing “what if” discussions, standardized patient visits which involve actors to simulate clinical conversations, part-task trainers which utilize anatomical models, screen-based simulations which involve interactive software-based computer patients, to high-fidelity simulations with replica of the clinical environments and settings (Gaba, 2007; Rosen, 2008). Simulations have been used to address aspects of the clinicians’ knowledge, skill, attitude, behavior, and other characteristics in training, human factors research, and performance assessment (Gaba, 2007; Weinger et al., 2017).

Simulation-based training enables the trainees to learn and practice patient care away from the bedside and without putting a patient at risk (Okuda et al., 2009). This is particularly important for crisis event management (CEM) training, which aims to prepare the trainees for dynamic decision-making and teamwork in high-risk, stressful situations in which the patient’s life is at stake (Gaba et al., 2001; Lighthall et al., 2003; Easdown et al., 2013). A randomized control trial showed that trainees who participated in high-fidelity simulation training performed better in real-life cardiopulmonary bypass than those who participated in traditional interactive seminar (Bruppacher et al., 2010).

During complex high-tempo, high-risk, and multi-person work, effective teamwork is essential to manage and monitor individual and team workload, allocate tasks, and maintain situation awareness. The ability of operating room (OR) teams (i.e., surgeons, anesthesia providers, nurses, techs, and other OR staff) to deliver high quality, safe care to patients depends on acting quickly and effectively, both individually and as a team. Effective training and evaluation must go beyond individual skills to include interactions among team members, and how those interactions transfer to operational environments. In addition, teamwork is a learned skill that can be improved with training (Easdown et al., 2013). The Anesthesia Crisis Resource Management curriculum emphasize the training of individuals to work in teams (Howard et al., 1992; Gaba et al., 2001).

### Measuring Performance and Experience in High-Fidelity Simulations

A recent review of simulation-based training research in healthcare concluded that outcome measurement was one of the greatest challenges in the field (McGaghie et al., 2010). There are four main methods to measure team performance and experience in high-fidelity simulations in healthcare: observer ratings, self-reports, behavioral performance measures, and neurophysiological indicators (McGaghie et al., 2010; Doumouras et al., 2012; Forsyth et al., 2017; Robertson et al., 2017). There are advantages and disadvantages for each method. Observer-rated measurement systems, such as the Anaesthetists’ Non-Technical Skills (ANTS) (Fletcher et al., 2003) and the Observational Teamwork Assessment for Surgery (OTAS) (Hull et al., 2011), usually require experienced clinicians or researchers to observe the participants’ behaviors, compare those behaviors with the provided behavioral markers, and produce standardized ratings. However, some systems are easier to use that others (Watkins et al., 2017) and these ratings are subject to potential biases from cognitive, social and environmental sources (Williams et al., 2003). Self-reported measures, such as the Surgery-specific Task Load Index, SURG-TLX (Wilson et al., 2011), and the Perceived Stress Scale (Cohen et al., 1983; Larkin et al., 2010), can provide an understanding of participants’ subjective experience. However, they have to be administered after the fact because except for very brief tools (e.g., the Borg workload scale; Weinger et al., 2004), completing them during a simulation can influence native task performance and even the domain being measured (e.g., workload) (Weinger and Slagle, 2002). Behavioral performance measures and neurophysiological indicators are usually technology-based assessment methods. Behavioral performance can be obtained through haptic sensors or optical systems embedded in the simulator (Rutherford et al., 2015). For example, D’Angelo et al. (2015) used optical motion tracking and analysis to capture the hand movement path length and suture time to show how participants improved their performance over time in a simulated suturing task. Neurophysiological indicators can measure participants’ cognitive states, such as attention and stress. For example, heart rate, respiration rate, and electrodermal activity can be used to measure stress levels (Rutherford et al., 2015). However, these methods are rarely used to measure team-level experience.

Feasible approaches to address the measurement issue in high-fidelity simulations are to integrate multiple measurement methods and to continue to develop new methods, especially technology-based assessment methods (Forsyth et al., 2017). Traditional physiological measures, such as cardiovascular and electrodermal activity measures, are influenced by cognitive, affective, physical movement, and other systemic physiological factors (Boucsein and Backs, 2000). As a result, it is difficult to establish their validity and sensitivity in realistic environments (Xu et al., 2017). Direct measurement of brain activity may be more useful in measuring cognitive status. Recent advances in neuroergonomics, which consider the neural mechanisms of human performance (Parasuraman and Mehta, 2015), and portable neurophysiological sensing technologies provided promising new methods to study human cognition and performance in realistic environments. One of those methods is to use functional near-infrared spectroscopy (fNIRS) to measure the participants’ level of brain activation as an indicator of workload and team engagement.

### Functional Near-Infrared Spectroscopy Measures

fNIRS technology infers changes of the oxygenated hemoglobin (HbO) and deoxygenated hemoglobin (HbR) levels in the cortical surface (Ferrari and Quaresima, 2012; Boas et al., 2014). fNIRS technology takes advantage of the fact that the human tissues are relatively transparent to near-infrared (NIR) light and the main absorption agent of NIR light in human brain is hemoglobin. Furthermore, HbO and HbR absorb NIRS light differently depending on the light’s wavelength. Thus, fNIRS technology is able to measure changes of HbO and HbR by shining NIR light with two or more wavelengths, detecting the reflected light, and quantifying the relative light attenuation. The measured changes of HbO and HbR levels can be related to neuronal activity in the corresponding region (Naseer and Hong, 2015). When a brain region is activated due to performance of relevant tasks, the cerebral blood flow (CBF) would increase to meet the metabolism requirement of the brain. While neural activity is associated with the conversion of HbO to HbR, the CBF increase provides an oversupply of HbO and “pushes out” HbR, thus leads to an increase of HbO and decrease of HbR in the brain region (Scholkmann et al., 2014).

Compared to other neural activity measures, such as electroencephalography (EEG) and functional magnetic resonance imaging (fMRI), fNIRS has unique advantages (Derosière et al., 2013). While hemodynamic signal measured by fNIRS is slower in detecting neural activation than the electrical signal measured by EEG, fNIRS provides better space localization capability and better resistance to muscular and movement artifacts than EEG (Naseer and Hong, 2015; Curtin and Ayaz, 2018). fNIRS has lower spatial resolution and penetration depth compared to fMRI, but it has higher temporal resolution; furthermore, the data can be captured using a portable form factor to enable studies in naturalistic environments (Balardin et al., 2017). For example, McKendrick et al. (2016) used a fNIRS system to evaluate portable and wearable device users’ prefrontal cortex (PFC) hemodynamics in outdoor navigation tasks. Yoshino et al. (2013) mounted a fNIRS device in a vehicle to study driver brain activation in actual highway driving.

fNIRS measures have been used as indicators of workload in both laboratory and applied settings. Laboratory studies have found that HbO increases and HbR decreases in the PFC region as workload increases in both n-back working memory tasks (Herff et al., 2014; Keshmiri et al., 2017) and mathematical tasks (Mandrick et al., 2013; Maior et al., 2014). In applied settings, fNIRS was used to measure workload in more complex tasks such as web form usability testing (Lukanov et al., 2016) and air traffic control (Harrison et al., 2014). Afergan et al. (2014) developed an adaptive system for unmanned aerial vehicles that can change task difficulty based on fNIRS-measured metrics of workload. However, few studies have used fNIRS measures as indicators of workload during acute event management in realistic environments.

The development of hyperscanning techniques, which enable the measurement of between-person brain activity dynamics (Scholkmann et al., 2013), facilitated research on groups or teams with fNIRS and other neural activity measures. Studies have investigated neural synchrony ranging from simple tasks, such as synchronizing button pressing or finger movements, to more complex ones, such as music production and trust games (Babiloni and Astolfi, 2014). Neural synchrony can be used as an indicator of the level of collective engagement in shared activities. For example, Osaka et al. (2015) found that pairs of singers showed a higher level of fNIRS neural synchrony in a cooperative singing condition than in a sing-alone condition. In face-to-face communication, Jiang et al. (2012) found that individuals showed higher level of fNIRS neural synchrony in conversations than in monologs; furthermore, Zhang et al. (2018) observed that in psychological counseling, client and counselor dyads showed higher levels of fNIRS neural synchrony during counseling than chatting. Other studies have used neural synchrony to predict team performance (Funane et al., 2011; Stevens et al., 2016). However, to our knowledge, no study to date has used fNIRS-derived neural synchrony to study crisis management teams in high-fidelity simulations.

### The Current Study

In the current study, we aimed to investigate the utilization of a portable fNIRS system to examine team experience in high-fidelity simulation-based CEM training for anesthesiologists in operating rooms. Specifically, we used PFC HbO and HbR levels as indicators of workload on the individual level, and neural synchrony as an indicator of team engagement on the team level.

**FIGURE 1:**
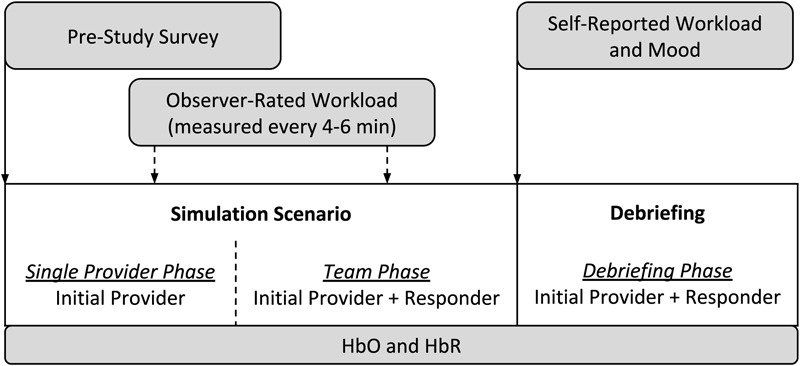
The process of a simulation session in the current study.

A typical simulation session consisted of a full-scale recreation of a challenging medical crisis situation using a computer-based patient mannequin, real clinical equipment and supplies, actors trained to portray other clinicians (e.g., surgeons, nurses, and technicians), and closely followed scripts, all set in a realistic clinical environment (McIvor et al., 2017; Weinger et al., 2017). Each simulation session consisted of two stages: the 10–20 min simulation scenario itself, which involved two and occasionally three trainees, and a typically 10–25 min instructor-facilitated debriefing, which included all of the trainees in the training session. During a simulation scenario, one trainee started the scenario on their own (the “initial provider”) while a second trainee (the “responder”) was sequestered in a quiet conference room to await being called to assist. Thus, data can be collected under three different conditions: single provider phase (i.e., initial provider only trainee in the simulation room, responder sequestered), team phase (both initial provider and responder in the simulation), and a debriefing phase. Figure 1 depicts the process of a typical simulation session.

Due to the structure of the simulation sessions and our review of the literature, we speculated that: (1) the workload of the initial provider (in simulation) should be higher than that of the responder (in sequestration) during the single provider phase; (2) the workload of the responder should be higher during the team phase than the single provider phase; and (3) the team should have more team engagement during the team phase (i.e., when working together) than during the single provider phase (not interacting). Therefore, we proposed the following hypotheses:

**H-1a**: The PFC HbO (HbR) level of the initial provider will be higher (lower) than that of the responder during the single provider phase of the scenario.
**H-1b**: The PFC HbO (HbR) level of the responder will be higher (lower) in the team phase than in the single provider phase of the scenario.
**H-2**: The team’s level of neural synchrony will be higher in the team phase than in the single provider phase of the scenario.

In addition to the hypotheses, we concurrently proposed a series of exploratory research questions (RQs) to guide our investigation of the use of fNIRS in the simulations. Specifically, we were interested in questions related to the sensitivity, convergent validity, and discriminant validity of PFC HbO and HbR as indicators of workload on the individual level:

**RQ-1a** (sensitivity): Is the PFC HbO (HbR) level sensitive to different levels of scenario difficulty?
**RQ-1b** (sensitivity): What is the pattern of PFC hemodynamics across different time segments in the scenario?
**RQ-1c** (convergent validity): Is there a significant correlation between the PFC HbO (HbR) and observer-rated or self-reported workload?
**RQ-1d** (discriminant validity): Is there a significant correlation between the PFC HbO (HbR) and self-reported mood?

We were also interested the sensitivity of PFC neural synchrony as an indicator of team engagement on the team level:

**RQ-2a** (sensitivity): Is the PFC neural synchrony level sensitive to different levels of scenario difficulty?
**RQ-2b** (sensitivity): What is the pattern of PFC neural synchrony across different time segments in the scenario?

## Materials and Methods

### Design

A typical CEM training session consisted of three simulation sessions involving two to four trainees. At any given training session, two to four trainees were enrolled in the study as participants. Most of the simulation scenarios involved two trainees and those were the target of our data collection. The two trainees were randomly assigned as initial provider and responder. All the scenarios included three phases: single provider, team, and debriefing. Each scenario followed one of the fifteen scenario scripts that required the trainees to manage a crisis event. For example, in the “Blown Intravenous (IV)” scenario, the trainees have to ascertain that their usual treatment (via IV drugs) of a deteriorating patient is not working, determine that it is due to an infiltrated IV, and institute alternative (non-IV) treatments. In the “ENT Airway Fire” script, the trainees have to manage a laser-instigated fire in the patient’s mouth and determine what to do next upon extinguishing it.

Trainees with different levels of experience went through different scenarios commensurate with their clinical experience. Scenario difficulty was adjusted to optimize learning (i.e., scenarios for experienced physicians were more difficult than were those for novice physicians). The relative difficulty levels of the scenarios were determined from ratings by three simulation instructors who were all experienced anesthesiologists and simulation instructors. All three instructors had all taught all of the studied scenarios. They rated each scenario’s difficulty on a five-point scale (independent of who might do the scenario). The intra-class correlation (ICC) among the ratings of the three instructors was 0.77, indicating an excellent level of inter-rater reliability (Hallgren, 2012). The rating for each scenario was then averaged across the three instructors. Finally, by applying the Jenks natural breaks classification method (Jenks, 1967) to the ratings, the scenario difficulty was categorized as low, medium, or high for the corresponding experience level of the trainees.

Thus, there were three independent variables (IVs): scenario phase (single provider, team, and debriefing), role (initial provider and responder), and scenario difficulty (low, medium, and high).

### Sample

This study was conducted in an academic medical center located in Nashville, TN, United States. The participants were resident and attending physicians in anesthesiology. An attending physician is a board-certified physician who can practice medicine independently. A resident physician is a medical school graduate who is training to become an attending physician. The participating residents were in the first, second, or third year of their residency training program (henceforth, they were referred as Y1, Y2, and Y3 residents). All participants were attending scheduled CEM training sessions at the time of study recruitment. The residents were required to attend the training while the attending physicians chose to do the training as part of their maintenance of certification in anesthesiology (MOCA). Table 1 provides a summary of the characteristics of the 33 individual participants. The study protocol was approved by the medical center’s Institutional Review Board and by the U.S. Army Medical Research and Materiel Command’s Office of Research Protections.

**Table 1:** Sample characteristics.

Experience level	Year 1 residents	Year 2 residents	Year 3 residents	Attending physicians	Total
Sample size	13	5	7	8	33
Age (years)	29.54 ± 2.47^a^	29.20 ± 1.64	30.14 ± 0.69	42.88 ± 6.08	32.85 ± 6.65
Gender (female)	3 (23.1%)	3 (60.0%)	3 (42.9%)	1 (12.5%)	10 (30.3%)
Prior residency training (months)	7.85 ± 3.44	25.00 ± 8.22	38.57 ± 5.86	–	19.88 ± 14.54
Prior clinical experience (years)^b^	–	–	–	9.88 ± 4.02	–
Prior times doing simulation training	2.54 ± 1.27	4.25 ± 0.50	11.00 ± 1.10	3.13 ± 6.88	4.55 ± 4.75
Prior night’s sleep duration (hours)	6.73 ± 1.02	7.35 ± 1.02	7.54 ± 1.81	7.37 ± 1.35	7.15 ± 1.30
Had difficulty falling asleep (“yes”)	4 (30.8%)	1 (20.0%)	3 (42.9%)	1 (12.5%)	9 (27.3%)
Had caffeinated drinks (“yes”)	11 (84.6%)	3 (60.0%)	5 (71.4%)	8 (100%)	27 (81.8%)

*^*a*^Data in this table are presented as Mean ± SD or Count (Percentage) when applicable. ^*b*^Years of experience since becoming an attending physician.*

A total number of 25 simulation sessions (11, 4, 5, and 5 for Y1 residents, Y2 residents, Y3 residents, and attending physicians, respectively) were included in the analysis. The number of different scenarios were 6, 2, 3, and 4 for Y1, Y2, and Y3 residents, and attending physicians, respectively.

### Procedure

Each participant was informed via email about the study opportunity prior to their scheduled training session. Upon arrival to the session, the study procedures and risks were explained, any questions were answered, and interested participants then signed a written informed consent document. Participants then filled out a pre-study survey that included questions about general demographics, clinical experience, and selected factors that could affect their performance and physiologic response to the stress of the training experience (Table 1). The baseline fNIRS measures were taken over a 5 min period where the participant sat still and filled out the pre-study survey.

During each scenario, using a tablet computer with a specialized software program, a trained research assistant (RA) noted any events of interest, including the start and end times of each phase of the scenario session. The RA also rated the initial provider’s workload (see section Workload and Mood) at 4–6 min random intervals as prompted by custom computer software. At the end of each scenario, all participants completed a survey to report their mood and workload during the simulation.

### Measures

#### fNIRS

We used the head-worn fNIRS Pioneer^TM^ sensor designed to be part of the MEDIC II system (Charles River Analytics, Cambridge, MA, United States)^1^ to collect HbO and HbR levels in the PFC. The device consisted of a head-worn sensor probe and a mobile-phone-sized hardware unit. The head-worn sensor probe, embedded in a flexible, black headband, included two light sources, which emitted infrared and visible light, and one light detector. The participants wore the sensor-embedded headband and then wore a surgical cap on top (all the trainees were required to wear surgical caps and scrubs during the CEM training). Pilot tests had demonstrated that this setup could hold the sensor probe stably in place and shield it from ambient light. The probe was secured without adhesion to the participant’s Fp2 region of the PFC, according to the international EEG 10-20 system (Klem et al., 1999). Thus, the fNIRS channel was located on the right side of the anterior PFC. Prior to placement, alcohol was used to clean the skin surface. The probe was connected via a thin cable to a lightweight hardware unit secured to the headband at the back of the participant’s head. The sensor recorded data at a sample rate of 100 Hz.

#### Workload and Mood

The Borg workload scale was the observer-rated workload measure. The Borg workload scale is a visual analog scale, ranging from 6 (no exertion) to 20 (maximum exertion), that has been shown to yield continuous parametric data (Borg, 1982) and has been used to assess health care providers’ overall workload in clinical settings (Weinger et al., 1994, 1997, 2004). The Borg scale correlates well with physiologic and other measures of workload and stress (Weinger et al., 2004).

The NASA Task Load Index (NASA-TLX) is a self-reported workload measure that includes six dimensions: mental demand, physical demand, temporal demand, performance, effort, and frustration (Hart and Staveland, 1988; Hoonakker et al., 2011). For each dimension, participants rated their experience during the scenario from 0 (“lowest”) to 100 (“highest”). An overall score was calculated by summing the ratings on the six dimensions.

Self-reported mood was measured using the Positive and Negative Affective Schedule (PANAS), a 20-item five-point Likert scale survey instrument (Watson et al., 1988; Crawford and Henry, 2004). A positive affect score and a negative affect score ranging from 10 to 50 were derived from participants’ ratings.

### Data Analysis

#### Case Segmentation

The cases were segmented to extract fNIRS data for subsequent analysis. The first kind of segments was the 1 min period immediately prior to each observer workload rating. The observer typically took less than 1 min to provide the workload rating once prompted by the computer software.

The second kind of segments involved different time points across a scenario. The different scenario phases (i.e., single provider, team, and debriefing) varied appreciably in duration across cases. For example, debriefings varied from 7 to 31 min in duration. Thus, to facilitate data analysis across cases, within each phase, we considered data at three time points (segments): the first minute, the middle minute, and the last minute. These were calculated as the 60 s period at the beginning, the middle, and the end of each scenario phase. The 1 min length was chosen to avoid overlapping time windows as well as provide consistency with the first kind of segmentation.

#### fNIRS Data Processing

The raw data from the MEDIC II fNIRS module included changes in optical density (OD) for two channels of light with different wavelengths. Changes in concentrations of HbO and HbR were calculated from the OD data using the modified Beer-Lambert Law (Cope and Delpy, 1988). We applied the wavelet-based motion artifact removal procedure with a tuning parameter value α = 0.15 to all HbO and HbR time series (Behnam and Guy, 2012; Cooper et al., 2012). We accounted for global drift (low-frequency noise) and high-frequency noise, by applying a band-pass third-order Butterworth filter with 0.01–0.1 Hz cutoff frequencies. After artifact removal and filtering, the data were visually inspected to ensure data quality; no manual corrections were made. The HbO and HbR data were then normalized within each participant. To express HbO and HbR data at any given moment as a relative change from the baseline, the mean HbO and HbR value during the baseline period was subtracted from all recorded data before further data processing and analysis. Data processing was performed in R (R Core Team, 2018) using the “signal” (Ligges et al., 2015) and “wavelets” (Aldrich, 2013) packages.

For the analysis at the individual level, mean levels of HbO and HbR were calculated for the time periods of interests. First, mean HbO and HbR were calculated for each of the three phases in each simulation session. Then, mean HbO and HbR were calculated for the first, middle, and last minute of each phase in a simulation session. In addition, mean HbO and HbR were calculated for the 1 min period immediately prior to each observer workload rating.

**FIGURE 2:**
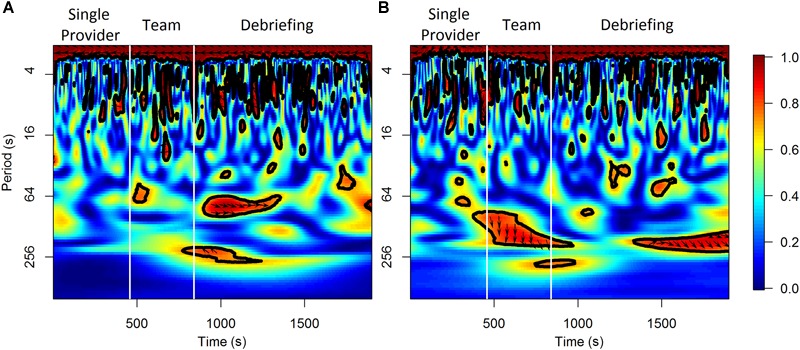
An illustrative example of the results of WTC analysis for HbO **(A)** and HbR **(B)** from a scenario session.

For the analysis on the team level, the wavelet transformation coherence (WTC) (Torrence and Compo, 1998; Grinsted et al., 2004) of the HbO and HbR between the two participants in a scenario session were calculated as indicators of neural synchrony (Cui et al., 2012; Osaka et al., 2015; Baker et al., 2016; Nozawa et al., 2016). WTC measures the cross-correlation of two time series based on the continuous wavelet transform at the given frequency and time. The HbO and HbR time series were re-sampled to 1 Hz for this analysis. The Morlet wavelet function with the parameter ω_0_ = 6 was used in the wavelet transformation. Only the coherence values outside of the cone of influence (COI) was considered to control the edge-effects of the WTC estimation. The frequency band to be considered in WTC varied in previous fNIRS hyperscanning studies depending on the task-related frequency band of each study, however, task-related bands are difficult to define for activities occur in natural and unstructured settings (Nozawa et al., 2016). This study selected a relatively wide frequency range of 0.01–0.1 Hz according to previous studies in natural settings (Jiang et al., 2012; Zhang et al., 2018). Mean coherence values at 0.01–0.1 Hz were calculated for the baseline and other time periods of interest (i.e., the 1 min periods prior to the observed workload ratings, the different scenario phases, and the three segments within each scenario phase). An illustrative example of the results of WTC analysis from one scenario session is shown in Figure 2. The final WTC value for each time period was calculated as the mean coherence value of that time period minus the mean coherence value of the corresponding baseline period. The WTC analysis was performed in R using the “biwavelet” package (Gouhier et al., 2018).

#### Statistical Analysis

The main statistical analysis was conducted using Bayesian linear mixed effects (LME) models with Markov Chain Monte Carlo (MCMC) estimation (Hadfield, 2010). The LME models accounted for the fixed effects of the IVs as well as the random effects of the participants, teams, or scenarios. Table 2 shows the model specifications for the LME models used in the analysis. The random effects were modeled as random intercepts. These statistical analyses were conducted using R with the “MCMCglmm” package (Hadfield, 2010). The MCMCglmm algorithm used non-informative priors and default parameter estimation settings (iterations = 13,000, thinning interval = 10, burn-in = 3,000). All the models were checked for convergence and autocorrelation of the estimates. An effect was considered significant if the 95% highest density interval (HDI) did not contain zero and the Bayesian “*p*-value” pMCMC was smaller than 0.05.

**Table 2:** LME model specifications.

Model ID	Dependent variable	Independent variables (fixed effects)	Independent variables (random effects)
1	HbO or HbR mean	• Scenario phase	• Participant
		• Role	• Team
		• Scenario difficulty	• Scenario
		• All possible interactions of the previous variables	
		• Experience level	
2	HbO or HbR mean	• Scenario phase (with first/middle/last minute)	• Participant
		• Role	• Team
		• Scenario difficulty	• Scenario
		• All possible interactions of the previous variables	
		• Experience level	
3	Observer-rated workload	• Scenario phase	• Participant
		• Scenario difficulty	• Team
		• All possible interactions of the previous variables	• Scenario
		• Experience level	
4	Self-reported workload, positive mood, or negative mood	• Role	• Participant
		• Scenario difficulty	• Team
		• All possible interactions of the previous variables	• Scenario
		• Experience level	
5	HbO or HbR WTC	• Scenario phase	• Team
		• Scenario difficulty	• Scenario
		• All possible interactions of the previous variables	
		• Experience level	
6	HbO or HbR WTC	• Scenario phase (with first/middle/last minute)	• Team
		• Scenario difficulty	• Scenario
		• All possible interactions of the previous variables	
		• Experience level	

The correlations between the dependent variables (DVs) were estimated using a multivariate multilevel modeling approach (Baldwin et al., 2014; Luo et al., 2015). The multivariate multilevel models were fitted using MCMCglmm. A correlation was considered significant if the 95% HDI did not contain zero.

## Results

### Individual Level Results

#### H-1a and H-1b Were Supported by HbR but Not HbO

**FIGURE 3:**
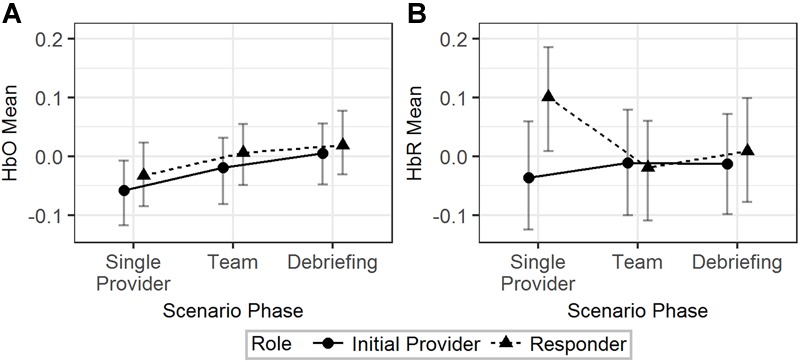
The predicted means and 95% HDIs of the initial provider and the responder’s HbO **(A)** and HbR **(B)** levels at different scenario phases. The values of all the other variables were held at their means.

The effects of scenario phase, role, and scenario difficulty on the mean HbO and HbR were tested using Model 1 (Table 2). The results are visualized in Figure 3. The difference of HbO mean between initial provider and responder in the single provider phase was not significant (*b* = 0.02, 95% HDI = [-0.04, 0.09], pMCMC = 0.43). The difference of the HbO mean of the responder between the single provider phase and the team phase was also not significant (*b* = 0.04, 95% HDI = [-0.01, 0.10], pMCMC = 0.18). HbR mean of the initial provider was significantly lower than the responder in the single provider phase (*b* = 0.14, 95% HDI = [0.02, 0.23], pMCMC = 0.01). The responder’s HbR mean was significantly lower in the team phase than in the single provider phase (*b* = 0.12, 95% HDI = [0.02, 0.20], pMCMC = 0.01).

In summary, **H-1a** and **H-1b** were both supported by the results from the HbR but not the HbO data.

#### The Effect of Scenario Difficulty (RQ-1a)

Scenario difficulty influenced the effect of scenario phases on HbO mean. When scenario difficulty was held at its mean, HbO mean was higher in the team/debriefing phases than in the single provider phase (*b* = 0.05, 95% HDI = [0.01, 0.09], pMCMC = 0.01) for both the initial provider and responder. This effect was smaller in the medium/high difficulty than in the low difficulty (*b* = -0.13, 95% HDI = [-0.21, -0.05], pMCMC < 0.01); so that the difference between the team/debriefing phases and the single provider phase was not significant in both medium difficulty (*b* = 0.003, 95% HDI = [-0.05, 0.06], pMCMC = 0.94) and high difficulty (*b* = 0.007, 95% HDI = [-0.05, 0.07], pMCMC = 0.85). These significant effects were not observed in HbR mean.

In summary, an HbO increase in team/debriefing phase compared to the initial provider phase was observed in the low difficulty scenarios but not in the medium/high difficulty scenarios. No scenario difficulty moderation effect was observed in the HbR data.

#### The Hemodynamics Across Time Segments (RQ-1b)

**FIGURE 4:**
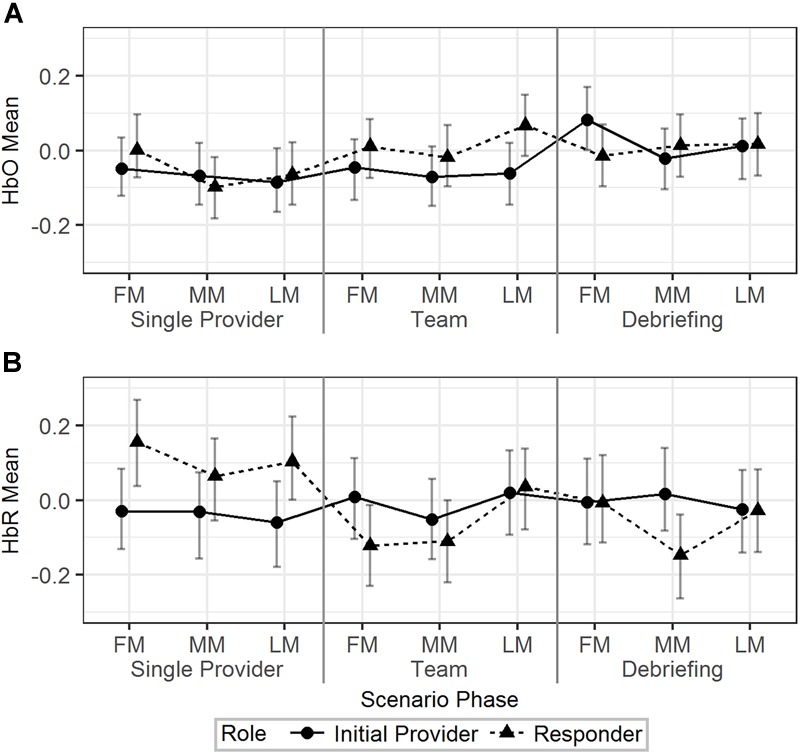
The predicted means and 95% HDIs of the initial provider and the responder’s HbO **(A)** and HbR **(B)** levels at the first/middle/last minutes (FM, MM, and LM) of the different scenario phases. The values of all the other variables were held at their means.

Model 2 (Table 2) was used to analyze the effect of scenario phase (using first, middle, and last minute) on HbO and HbR. The results are visualized in Figure 4. For HbO, a small but significant increase was observed at the last minute of the team phase compared to the last minute of the single provider phase (*b* = 0.11, 95% HDI = [0.03, 0.19], pMCMC < 0.01). There was a significant interaction effect that the initial providers’ HbO increased in the first minute of the debriefing phase compared to the last minute of the team phase, while the responders’ HbO decreased (*b* = 0.23, 95% HDI = [0.07, 0.37], pMCMC < 0.01). Responders showed higher levels of HbR than the initial providers in the first (*b* = -0.18, 95% HDI = [-0.33, -0.03], pMCMC = 0.02) and last (*b* = -0.16, 95% HDI = [-0.31, -0.01], pMCMC = 0.04) minutes of the single provider phase. The HbR level of the responder decreased in the first minute of the team phase compared to the last minute of the single provider phase, while the HbR level of the initial provider increased in the same period (*b* = 0.29, 95% HDI = [0.09, 0.49], pMCMC < 0.01).

In summary, the hemodynamics analysis across time segments revealed additional information regarding the HbO/HbR change in different time points across the scenarios and the different patterns of HbO/HbR change related to different roles.

#### The Correlations Among HbO, HbR, Workload, and Mood (RQ-1c and RQ-1d)

The effects of the IVs on the observer-rated workload and self-reported workload and mood were first tested. The results from Model 3 (Table 2) indicated that there was no significant effect of scenario phase and scenario difficulty on observer-rated workload of the initial provider. Model 4 (Table 2) was used to explore the effects of role and scenario difficulty on self-reported workload and mood. No significant effect of the IVs on self-reported workload or negative mood was observed. Initial providers reported higher positive mood than responders in low difficulty scenarios (*b* = 10.95, 95% HDI = [3.75, 17.29], pMCMC < 0.01).

A multivariate model based on Model 3 was fitted to the data to explore the correlations between HbO/HbR and observer-rated workload. Mean levels of HbO and HbR were calculated for 1 min prior to the moment the observer-rated workload rating was given. The correlations were not significant (-0.05 < *r* < 0.05). Correlations among HbO, HbR, and self-reported workload and mood were, similarly, tested based on Model 4. Using mean levels in the team phase, HbO and HbR correlated poorly with the self-reported metrics (-0.05 < *r* < 0.05). The only significant correlation was between self-reported workload and negative mood (*r* = 0.39, 95% HDI = [0.07, 0.66]).

In summary, HbO/HbR were not significantly correlated with the workload and mood measures.

### Team Level Results

#### H-2 Was Supported by Both HbO and HbR WTCs

Model 5 (Table 2) was fitted to the data to test the effects of scenario phase and difficulty on HbO and HbR WTCs. WTC was higher in the team phase than in the single provider phase for both HbO (*b* = 0.08, 95% HDI = [0.06, 0.11], pMCMC < 0.01) and HbR (*b* = 0.08, 95% HDI = [0.05, 0.10], pMCMC < 0.01). Also note that HbO WTC was significantly higher in the team phase than in the debriefing phase (*b* = 0.04, 95% HDI = [0.01, 0.07], pMCMC < 0.01), while the same effect was not significant for HbR WTC (*b* = 0.02, 95% HDI = [-0.01, 0.04], pMCMC = 0.17).

In summary, **H-2** was supported by findings from both HbO and HbR data.

#### The Effect of Scenario Difficulty (RQ-2a)

**FIGURE 5:**
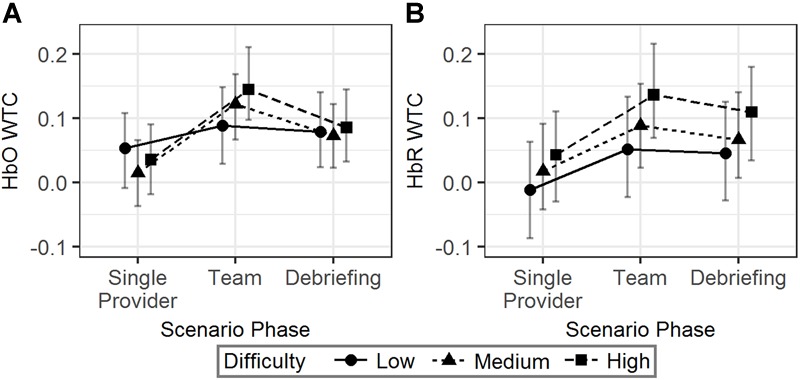
The predicted means and 95% HDIs of the WTCs of HbO **(A)** and HbR **(B)** between the initial provider and the responder in a team at different scenario phases. The values of all the other variables were held at their means.

The interaction effect showed that the HbO WTC difference between the single provider phase and the team phase was larger in the high difficulty scenarios than in the low difficulty scenarios (*b* = 0.07, 95% HDI = [0.01, 0.14], pMCMC = 0.04). No significant effect associated with scenario difficulty was observed for HbR WTC. The results are visualized in Figure 5.

In summary, more difficult scenarios were correlated with an increased HbO WTC in team phase compared to the single provider phase. However, similar effects were not observed in HbR WTC.

#### The Neural Synchrony Across Time Segments (RQ-2b)

**FIGURE 6:**
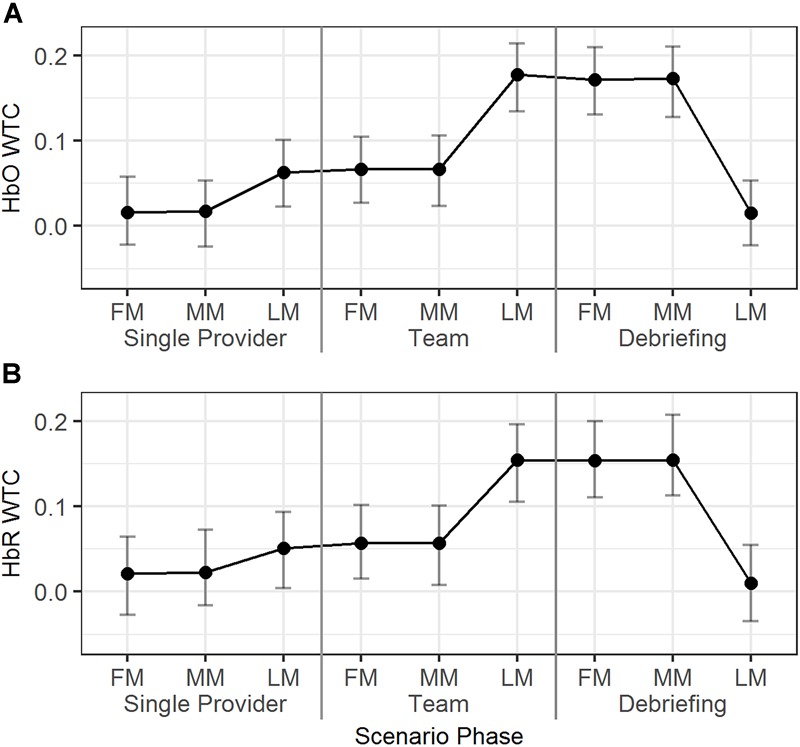
The predicted means and 95% HDIs of the WTCs of HbO **(A)** and HbR **(B)** between the initial provider and the responder in a team at first/middle/last minutes (FM, MM, and LM) of the different scenario phases. The values of all the other variables were held at their means.

The temporal dynamics of the HbO/HbR WTCs in the first, middle, and last minutes of the different scenario phases were explored using Model 6 (Table 2). As seen in Figure 6, for HbO, the WTC level increased in the last minute compared to the middle minute of the single provider phase (*b* = 0.05, 95% HDI = [0.01, 0.08], pMCMC = 0.02). The WTC also increased in the last minute compared to the middle minute of the team phase (*b* = 0.11, 95% HDI = [0.08, 0.15], pMCMC < 0.01). WTC then decreased in the last minute compared to the middle minute of the debriefing phase (*b* = -0.16, 95% HDI = [-0.19, -0.12], pMCMC < 0.01). The HbR WTC showed a similar pattern; there was a significant increase in the last minute compared to the middle minute of the team phase (*b* = 0.10, 95% HDI = [0.06, 0.14], pMCMC < 0.01) and a decrease in the last minute compared to the middle minute of the debriefing phase (*b* = -0.15, 95% HDI = [-0.18, -0.11], pMCMC < 0.01).

In summary, dramatic HbO/HbR WTC increases were observed around the end of the team phase and the high WTC levels maintained until around the end of the debriefing phase.

## Discussion

The current study used a portable fNIRS system, the fNIRS Pioneer^TM^, to measure PFC hemodynamics in clinicians doing CEM high-fidelity simulations. On the individual level, we found that the pattern of HbR mean for the different roles in different scenario phases was consistent with our hypotheses while HbO mean was not. However, HbR mean was not sensitive to different levels of scenario difficulty. Furthermore, the HbO and HbR means were not significantly correlated with any of the observer-rated or self-reported workload and mood measures. The teams showed higher HbO/HbR neural synchrony during periods of teamwork, supporting our hypothesis. In addition, HbO neural synchrony was sensitive to different levels of scenario difficulty; higher level of neural synchrony was observed in scenarios with higher level of difficulty. Analysis of the time segments indicated that the neural synchrony of the teams increased dramatically in the last minute of the team phase compared to previous segments, and this higher level was maintained until the end of the debriefing phase.

### fNIRS Measured Brain Activity as Indicator of Workload for Individual Clinicians

#### fNIRS Measures in Different Scenario Phases

The findings suggest that HbR may be a better indicator for workload than HbO in the current study setting. Regarding the use of HbO vs. HbR, some prior studies found that HbR was more sensitive than HbO (e.g., Peck et al., 2013), however, some studies have reported that HbO and HbR are, similarly, sensitive to changes in workload (e.g., Herff et al., 2014; McKendrick et al., 2016) while others have suggested that HbR is *less* sensitive than HbO (e.g., Derosière et al., 2015). In the brain-computer interface studies, HbR was less sensitive for real time cortical activation detection (Naseer et al., 2014) and HbO provided better test-retest reliability (Plichta et al., 2006). Overall, the results presented in the literature are not conclusive.

Physiologically, increased cognitive workload typically associates with increased activation in the PFC region (Parasuraman and Caggiano, 2005), as a result, one should observe an HbO increase and an HbR decrease in high workload conditions compared to low workload conditions. However, fNIRS measures are influenced not only by the neurovascular coupling which reflects cerebral neuronal activity, but also systemic physiological changes in the cerebral and extracerebral compartments (Tachtsidis and Scholkmann, 2016). Studies have found that anesthesiologists’ workload correlates with systemic physiological changes, such as heart rate and heart rate variability (Weinger and Slagle, 2002; Weinger et al., 2004). Thus, in such a study setting, as HbR may be less influenced by global hemodynamic changes (Kirilina et al., 2012), when the signal-to-noise ratio is optimized, it can be a reliable indicator for task-related cortical activation (Piper et al., 2014).

#### fNIRS Measures in Scenarios With Different Difficulty Level

While HbR level was able to differentiate periods of resting (i.e., responder in the single provider phase) and working (i.e., responder in the team phase and initial provider in the single provider phase) and revealed an interesting pattern across time segments within a scenario, it was not sensitive to scenario difficulty. It was assumed that the more difficult a scenario was, the more cognitive resource the participants must use to work. In more difficult scenarios, the participants’ PFC may activate to a greater extent or for longer durations. As a result, the mean HbO level should be higher and the mean HbR level should be lower over the scenario period. For example, studies have found the associations between fNIRS measured hemodynamics and different levels of task difficulty in driving (Foy and Chapman, 2018) and flight (Gateau et al., 2015). However, anesthesiology work involves more physical activities, which introduces additional systemic physiological changes, than driving and flight. More research is needed to improve the sensitivity and specificity of the fNIRS measures before they will be useful for evaluating workload in this domain.

#### Correlations With Other Measures of Workload

In the present study, neither HbO nor HbR correlated with observer-rated or self-reported workload. Previous studies have found that different workload measures are sensitive, but they have poor correlation with each other (Myrtek et al., 1994; Matthews et al., 2015). Workload is a multidimensional construct; therefore, different measures may reflect different dimensions of workload (Matthews et al., 2015; Young et al., 2015). At the same time, different measurement methods may provide unique advantages in certain situations. Based on changes in HbR levels, this study found that the initial providers’ workload decreased in the transition between the single provider phase and the team phase, while the responders’ workload increased during the same time period. These types of dynamic changes would be very difficult to capture with self-reported workload measures.

### Neural Synchrony in Anesthesiology Teams

#### fNIRS Neural Synchrony as a Measure of Team Engagement

The results from the team level neural synchrony analysis showed that fNIRS measures are feasible and sensitive indicators of team engagement. Both HbO and HbR WTCs were able to differentiate periods of individual work (i.e., single provider phase) from teamwork (i.e., team phase). These findings are consistent with previous study results regarding cooperative work in other settings, such as laboratory n-back task (Dommer et al., 2012) and singing (Osaka et al., 2015), where significantly higher levels of fNIRS PFC neural synchrony were observed in cooperative work compared to individual work.

Furthermore, HbO WTC was sensitive to changes in scenario difficulty. More difficult scenarios required closer cooperation between the team members compared to less difficult ones, thus the team members might have shown a higher level of engagement in teamwork. The increased level of team engagement corresponded to an increased level of neural synchrony. For example, Toppi et al. (2016) have found that pairs of pilots showed an increased level of EEG neural synchrony in the frontal and parietal brain areas in more cooperation-demanding task scenarios in simulated flight. Zhang et al. (2018) found that the fNIRS neural synchrony in the right temporo-parietal junction area between psychological counselors and clients increased as the dyads increased their engagement in the conversation. This study demonstrated that fNIRS neural synchrony can be useful in detecting team engagement levels in anesthesiology teams. In practice, fNIRS neural synchrony may be useful as a quantitative guide to the design of simulation scenario difficulty to fit trainee expertise level. Note that although HbO was less sensitive than HbR in measuring workload (possibly due to systemic physiological noise), it was more sensitive in measuring team engagement. Future research should investigate how systemic physiological noise may influence WTC and why HbR was a less sensitive team engagement measure.

#### Temporal Dynamics of Neural Synchrony in Anesthesiology Scenarios

This study revealed interesting patterns in the level of neural synchrony across different time segments within a simulation session. First, there was a dramatic increase in neural synchrony at the end of the team phase, when the teams were actively coordinating to implement treatment in the face of a crisis situation. Simulation scenarios are written to allow the instructor to conclude when the team has achieved key treatment milestones. Thus, one might postulate that the team neural synchrony is an indicator of a coherent team mental model that either allowed or was a consequence of successful interventions. Future studies should examine team neural synchrony in successful vs. unsuccessful event management. Second, there was a sustained level of neural synchrony throughout most of the debriefing. One of the main goals of instructor-facilitated debriefing is to achieve a common understanding (i.e., common ground) of what happened during the scenario. The debriefing is a period of high participant engagement in the discussion regarding what happened and why, and how performance could be improved. Third, the level of neural synchrony decreased in the last segment of the debriefing phase when the group was wrapping up the discussion and either preparing for the next simulation session or concluding the day’s training.

### Limitations

This study has limitations. First, the study sample size was small due to limited supplies of clinicians and other logistical and technological constraints. As a result, we were not able to directly compare clinician groups of different expertise levels and had to control their effects statistically. Second, the three 1 min time points in a scenario phase may not have provided enough information on the temporal dynamics of HbO/HbR means and WTCs. Third, due to logistic restrictions, baseline fNIRS measure was collected when the participants were filling out surveys. Compared to a traditional resting baseline (Fishel et al., 2007), our baseline involved a certain degree of cognitive activity. Individual variations in cortical activation during the baseline might have decreased the power to detect significant changes over time. Fourth, the portable fNIRS system used in the current study only provided one channel of HbO/HbR data from one light source – detector pair. Without multiple data channels, we had to use univariate methods to process the fNIRS data which is not as robust as multivariate methods in removing artifacts from sources such as motion and physiological noise (Scholkmann et al., 2014; Naseer and Hong, 2015).

## Conclusion

In this paper we studied a portable fNIRS system, the fNIRS Pioneer^TM^, to measure team experience in high-fidelity CEM clinical simulation. We found that HbR level may be a useful indicator of workload on the individual level, but its sensitivity needs further study. Neural synchrony based on HbO/HbR WTCs appears to be a promising fNIRS measure of team engagement. The use of a portable fNIRS system in CEM is feasible but challenging. In practice, fNIRS may best be used in combination with other physiological and psychological (i.e., observer ratings and self-reports) measures, to provide a comprehensive perspective on team experience and performance. Specific direction for future studies include: (1) test the use of fNIRS in CEM simulation with a larger sample size to compare the effect of clinician expertise; (2) compare neural synchrony measures with other team level measures, such as observer-rated and self-reported teamwork and team performance; (3) improve the design of the portable fNIRS system to achieve a balance of size, weight, performance (e.g., more available data channels), and usability in realistic high-tempo practice environments.

## Data Availability

The datasets generated for this study are available on request to the corresponding author.

## Ethics Statement

This study was carried out in accordance with the recommendations of Vanderbilt Human Research Protection Program policies and procedures, Behavioral Sciences Committee – Institutional Review Board, with written informed consent from all subjects. All subjects gave written informed consent in accordance with the Declaration of Helsinki. The protocol was approved by the Vanderbilt University Behavioral Sciences Committee – Institutional Review Board.

## Author Contributions

JX, JS, AB, BB, and MW designed the research. JX and JS performed data collection with the assistance from AB and MW. JX analyzed the data and drafted the manuscript. BB provided technical support for data collection and analysis. The other authors revised the manuscript.

## Conflict of Interest Statement

The authors declare that the research was conducted in the absence of any commercial or financial relationships that could be construed as a potential conflict of interest.

## Footnotes

1 http://biosignalsplux.com/en/fnirs-sensor
